# Regulatory T cells in inflammation and resolution of acute lung injury

**DOI:** 10.1111/crj.13527

**Published:** 2022-08-04

**Authors:** Linlin Wang, Weipeng Jiang, Xiaocen Wang, Lin Tong, Yuanlin Song

**Affiliations:** ^1^ Department of Pulmonary Medicine, Zhongshan Hospital Fudan University Shanghai China; ^2^ Shanghai Key Laboratory of Lung Inflammation and Injury, Department of Pulmonary Medicine, Zhongshan Hospital Fudan University Shanghai China; ^3^ Shanghai Institute of Infectious Disease and Biosecurity Shanghai China; ^4^ Shanghai Respiratory Research Institute Shanghai China; ^5^ National Clinical Research Center for Aging and Medicine, Huashan Hospital Fudan University Shanghai China; ^6^ Jinshan Hospital of Fudan University Shanghai China

**Keywords:** ALI, ARDS, inflammation, regulatory T cells, resolution

## Abstract

**Introduction:**

Acute respiratory distress syndrome (ARDS) is characterized by hypoxemia and increased lung permeability and would result in acute respiratory failure and with high mortality. In patients who survive from acute lung injury (ALI)/ARDS, it is an active process of the transition from injury to resolution depending on the coordinated immune system. The roles of regulatory CD4^+^T cells (Tregs) are now gradually being clarified during inflammation and resolution of ARDS. However, clear conclusions about roles of Tregs in ALI/ARDS are only a few.

**Objective:**

This review provides an overview of phenotype, differentiation, and suppressive mechanisms of Tregs and focuses on keys of biology of Tregs in alveolar space during the inflammatory response and resolution of ALI/ARDS.

**Data Source:**

Literature search of Web of Science, PubMed, and EMBASE was made to find relative articles about Tregs in ALI/ARDS. We used the following search terms: Tregs, ALI, ARDS, inflammation, and resolution.

**Conclusion:**

More and more studies have indicated Tregs involved in the processes of inflammation and resolution of ALI/ARDS. A deep understanding of the roles of Tregs may indicate new treatments for patients of ARDS. Therapies aimed at expansion or adaptive transfer of Tregs could be an effective therapy to ARDS patients.

## Graphical abstract

The roles of regulatory CD4^+^T lymphocytes (Tregs) are now increasingly being recognized during resolution of ARDS. A dissection of the roles of Tregs may indicate the design of new treatments for ARDS patients. Therapies aimed at expansion or adaptive transfer of Tregs could be an effective therapy to ARDS patients.

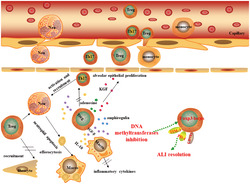

## 1 INTRODUCTION

The acute onset of ALI and ARDS is mainly manifested by respiratory distress and refractory hypoxemia with high mortality and poor prognosis. Now, the main clinical treatment methods are supportive treatment. Except for small tidal volume mechanical ventilation and prone position ventilation,^1^ which can reduce the mortality rate of ALI/ARDS, there are no other effective drug treatment measures. The existing schemes cannot achieve satisfactory results, and the mortality is still up to 40%.^2^ In addition to the above treatments, research on regulating the immune response of ALI/ARDS is also receiving increasing attention. Studies indicated that the recovery of lung injury depends on highly coordinated immune system.^3^, ^4^, ^5^, ^6^, ^7^, ^8^ In one our retrospective study, we found NLR (neutrophil and lymphocyte ratio) was significantly correlated with the prognosis of the ARDS. The prognosis was poor when the ratio was greater than 14, indicating that lymphocytes may be play an important role in the onset and development of ARDS.^9^

At present, the most widely studied lymphocyte subsets in ALI/ARDS are regulatory T cells (Tregs). In alveolar space, the processes of inflammation and resolution are closely related to the subpopulations and functions of Tregs. Researchers have identified the phenotype, mechanisms, and signal pathways of Tregs involved in acute inflammation and resolution in ALI/ARDS. Now, we will focus on keys of Tregs biology (subpopulations, differentiation, and function) of alveolar space during the inflammatory response and resolution of ALI/ARDS and explore new areas of therapeutic potential of ARDS.

## 2 PHENOTYPE AND DEVELOPMENT OF Tregs

Tregs play an important role during the suppression of immune response through different mechanisms.^10^, ^11^ Tregs mainly include two subgroups: nTregs (thymus‐derived Tregs) and iTregs (induced Tregs), which peripherally antigen‐induced Tregs generated in the periphery from naive CD4^+^T cells under certain antigenic stimuli or suppressor cytokines. iTregs are usually more plastic than nTregs.^12^ Although two Treg subpopulations exhibit different developmental mechanisms, they have a synergetic effect to maintain immune homeostasis and share similar phenotypes and suppressive function.

**FIGURE 1:**
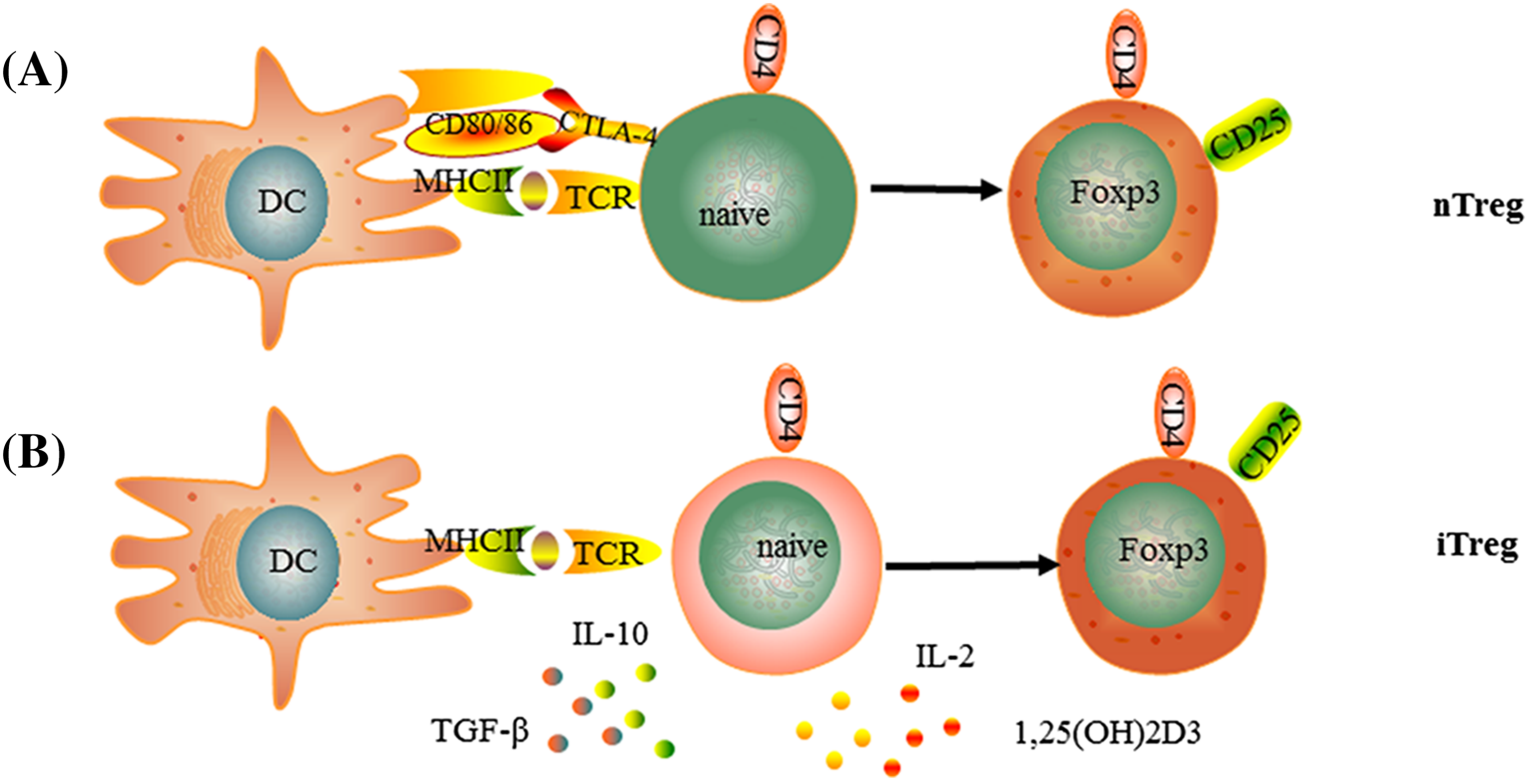
(A) nTreg develops in thymus. A strong TCR signal associated with CTLA‐4/CD80/CD86 upregulates CD25 in naïve CD4+T cells. Then, signals through CD25 lead to the expression of Foxp3. (B) The regulatory phenotype of iTreg induced in peripheral lymphoid organs stimulated by antigens and suppressive cytokines

The precursors that express TCRs with high affinity for self‐antigens can develop into nTregs.^13^ nTreg differentiation in thymus consists of two steps. The combination of a strong TCR signal with costimulatory molecules result in the upregulation of CD25 of naïve CD4+ T cells. Then, signal transducer and activator of transcription 5 (STAT‐5) are the downstream of CD25, can bind a regulatory sequence in the Foxp3 gene, and then promote Foxp3 expression, which is a necessary marker during the development and function of Tregs in the thymus and peripheral lymphoid organs,^14^, ^15^, ^16^ and Foxp3 locus conserved noncoding regions (CNS) of the regions also involved in Treg induction and stability (Figure 1A).^17^ After generation in thymus, nTregs migrate to the periphery to perform their suppressive function.^18^

nTregs express constitutively CD4 and CD25, but CD25 is also upregulated in effector T cells (Teffs) when activated. nTregs also constitutively express CTLA‐4 (cytotoxic T Lymphocyte antigen 4), CD62L, CD103, and GITR (glucocorticoid‐induced tumor necrosis factor receptor related protein), but expression of these markers is also affected by T cell activation and do not provide more specificity than CD25.^10^, ^19^, ^20^, ^21^ CD127 have been thought to be characteristic of nTregs,^22^ but its expression is downregulated after Teff activation.^23^ Thus, CD127 is only useful to identify Tregs in non‐inflammatory conditions. nTregs also express some non‐specific makers such as LAG‐3 (protein lymphocyte activation gene 3) and TLRs 4, 5, 7, and 8.^24^

iTregs express different levels of CD25. Most of iTregs highly express CD25, and a small group expresses small amounts of CD25, but both populations express Foxp3 (Figure 1B). iTregs are generated in the periphery induced from naive CD4^+^T cells with anti‐inflammatory cytokines and dendritic cells (DCs). A high concentration of TGF‐β is a critical cytokine for the generation and phenotype in T cells.^25^, ^26^, ^27^ TGF‐β affected the peripheral pool of Tregs,^28^ and the role of TGF‐β in development of iTregs was initially refuted. However, recent studies have showed that TGF‐β is necessary requirement for the generation of iTregs, indicating that TGF‐β also can induce Foxp3 expression of thymic Treg precursors in the context of TCR.^29^ Tolerogenic CD103^+^DCs can generated RA (retinoic acid) and 1,25(OH)2D3 (1,25‐dihydroxyvitamin D3) to provide a favorable environment for iTregs differentiation.^30^, ^31^, ^32^ 1,25(OH)2D3 combined with IL‐2 can induce both CTLA‐4 and Foxp3 expression of iTregs.^33^ The PD‐1/PD‐L1 signaling also plays critical roles in the generation, homeostasis, and plasticity of Foxp3^+^iTreg.^34^, ^35^, ^36^, ^37^, ^38^ The most common subgroups of Tregs include Tr1 cells (Type 1 regulatory T) and Th3 cells. Tr1 cells express low and transient levels of Foxp3 and secretion of high amounts of IL‐10, which induce anergy and low cell proliferation.^39^ They also produce IFN‐γ, TGF‐β, and IL‐15 and low levels of IL‐2 and IL‐4 and induce anergy and low cell proliferation by secretion of IL‐10.^39^, ^40^ There is no specific marker for Th1 cells, although some research has shown that GATA is a potential candidate.^41^ Th3 cells originate from CD4+T cells stimulated by TGF‐β and play critical role in oral tolerance by secretion of TGF‐β and IL‐10.^42^

Both nTregs and iTregs express CD25 and Foxp3. Thus, it remains challenging to distinguish these two subsets. Helios, Nrp‐1 (neuropilin‐1), and FR4 (folate receptor 4) are candidates for this discrimination. But these are no specific markers to distinguish nTregs from iTregs. Although nTregs can distinguish from iTregs by higher expression of Helios, a small group of human nTregs does not express Helios.^43^, ^44^ Furthermore, recent studies have reported that iTregs could also express Helios.^45^ Although nTregs exclusively express Nrp‐1, Nrp‐1 can also be induced in activated Teffs in humans.^46^, ^47^ High amounts of FR4 are found to express constitutively on nTregs, but it also expressed by iTregs.^48^ Thus, the contribution of nTregs and iTregs to the disease would be limited by the lack of definite markers, particularly in humans.

## 3 SUPPRESSIVE MECHANISMS OF Tregs

TCR repertoires of nTregs and iTregs are different leads to their function are distinct. iTregs are mainly involved in the tolerance to non‐self‐antigens while nTregs are preferentially responsible for control of auto‐specific responses.^49^ Tregs can regulate both innate and adaptive immune cells in various pathophysiological microenvironment though different suppressive mechanism.

Two main types of suppressive mechanisms of Tregs are contact‐dependent and contact‐independent. Tregs can regulate maturation and function of APC (antigen presenting cell) through the interaction of CTLA‐4, Nrp‐1, and LAG‐3 expressed on Tregs with the CD80/86 costimulatory molecules, MHC class II, and Sema4a expressed on APC.^50^, ^51^, ^52^ Tregs can also induce direct killing of Teffs through interaction of Gal‐9 expressed by Tregs and Tim‐3 expressed by Teffs. Tregs can express CD39/CD73 ectoenzymes to cleavage of ATP into adenosine.^53^, ^54^ Interaction of adenosine with the A2A receptor increases cAMP levels of target cells, thus inhibit cell proliferation of these target cells.^55^, ^56^, ^57^ Adenosine combined with A2A receptor expressed on Tregs can improve expression of Foxp3 and Tregs function.

**FIGURE 2:**
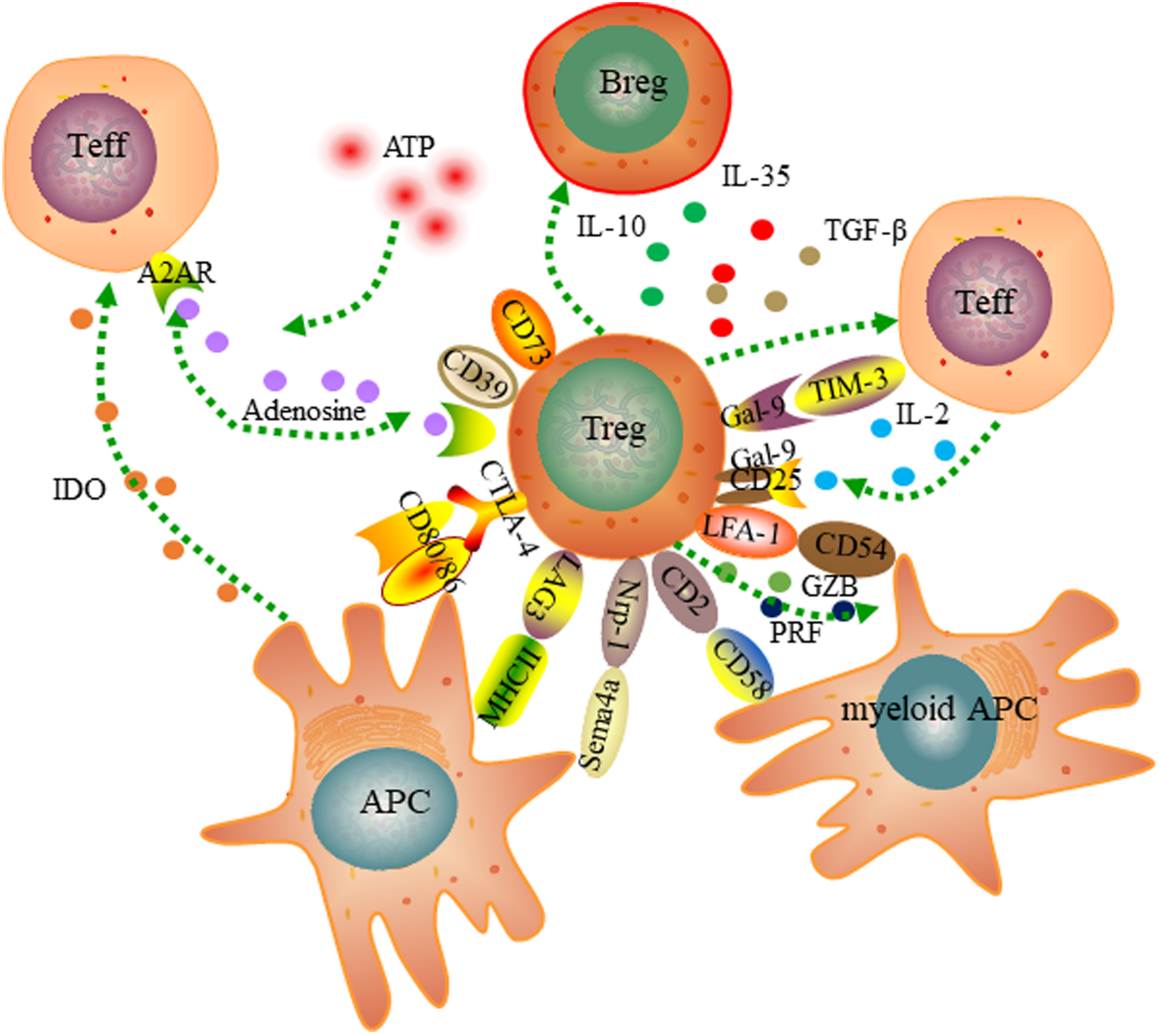
Tregs regulate immune responses through multiple suppressive mechanisms. Tregs can inhibit Teffs through the suppressive cytokines IL‐10, IL‐35, and TGF‐β. Tregs can disrupt metabolic functions of Teff through co‐expressing CD39/CD73 generating adenosine or IL‐2 deprivation. Interaction of CTLA‐4, LAG‐3, and Nrp1 expressed by Tregs with CD80/86 costimulatory molecules and Sema4a and MHCII expressed by APC can suppress maturation and function of Tregs, lead to production of IDO, and finally prevent maturation of APC maturation and activation of Teffs. Tregs also can induce direct killing of Teffs through the production of GZB or the interaction between Gal‐9 expressed by Tregs with Tim‐3 expressed by Teffs. GZB also can lyse myeloid APC through the interaction of CD2 and LFA‐1 expressed by Tregs and CD58 and CD54 expressed by myeloid APC.

Tregs can also secret anti‐inflammatory cytokines, such as TGF‐β, IL‐10, and IL‐35. These immunosuppressive cytokines in turn induce the development of iTregs. TGF‐β can inhibit the proliferation and differentiation of Th1 and Th2 by downregulating of the transcription of T‐bet and GATA‐3.^58^, ^59^ High level of TGF‐β also affects Th17 cells function.^27^ IL‐10 can suppress T cells responses by downregulating of IFN‐γ, IL‐2, and GM‐CSF.^60^ Furthermore, IL‐10 can induce IgG4 and suppress IgE of B cells to induce immune tolerance.^61^ IL‐35 is a novel anti‐inflammatory cytokine specifically secreted by Tregs, and necessary for maximal suppressive function of Tregs, can induce the development of iTregs, suppress the proliferation of Th1 and Th17 cells by inhibiting the G1 phase of cell division of early T cell rest,^62^ and inhibit development and proliferation of Th2 by repressing GATA3 transcription and IL‐4 secretion.^63^ IL‐35 can also regulate the plasticity of Th2, mediate differentiation of Th2 cells to Tregs (Figure 2).^64^

IL‐2 is required to expand Tregs and to induce their suppressive function in vitro. However, in vivo, Tregs are strong competitors for IL‐2 compared with their target cells via constitutively expressing CD25. Lack of IL‐2 causes apoptosis of target cells through the Bcl‐2/Bim and independently of PRF/Fas signaling pathway (Figure 2).^65^ Mice deficient in IL‐2 develop an unstable population of Tregs and subsequently acquire lymphoproliferative disease.^66^ From this side, it is difficult to reconcile with the idea that IL‐2 absorption plays a relevant role as an effector mechanism in vivo. Recent studies have proposed that Tregs can induce direct killing of Teffs through the GZB (granzyme B) production or by the interaction of Gal‐9 (galectin 9) expressed by Tregs with Tim‐3 (T‐cell immunoglobulin and mucin domain‐containing protein 3) expressed by Teff. GZB also can lyse myeloid APC through the interaction of CD2 and LFA‐1 (lymphocyte function‐associated antigen‐1) expressed by Tregs with CD58 and CD54 expressed by myeloid APC (Figure 2).^67^

## 4 TREGS IN ACUTE LUNG INJURY

ARDS is a fatal inflammatory lung disease with high mortality without effective therapies. More and more studies have shown that Tregs resolve inflammation of lung, but mechanisms of Tregs to promote the resolution of lung injury remain unknown. Investigators are ongoing into whether the known mechanisms of suppressive function of Treg are effective in ALI/ARDS. Presumably, if we could understand how Tregs play roles and change in ALI/ARDS, then effective therapies could be designed to treat ALI/ARDS by regulating the number and function of Tregs either endogenously or exogenously.

In Tregs, CD4^+^CD25^+^Foxp3^+^Tregs are the most studied in ALI/ARDS. D'Alessio et al. first show that CD4^+^CD25^+^Foxp3^+^Tregs could mediated the active resolution of ALI mouse model.^8^ Many studies have indicated that CD4^+^CD25^+^Foxp3^+^Tregs are associated with severity of ALI/ARDS. Adamzik group found CD4^+^CD25^+^Foxp3^+^Tregs could be activated in ARDS patients and increased in alveola and even could predict poor outcome of ARDS.^68^ Sebastien et al. also found that the quantity and function of Tregs changed in ARDS patients.^69^ A prospective, observational study performed by Yu group indicated ratio of Th17/Treg ratio >0.79 was the independent predictor for 28‐day mortality in ARDS patients.^70^ Our recent studies also found there was imbalance of Tregs and Th17 cells and Tregs/Th17 ratio downregulated in LPS‐induced ALI model.^71^

**FIGURE 3:**
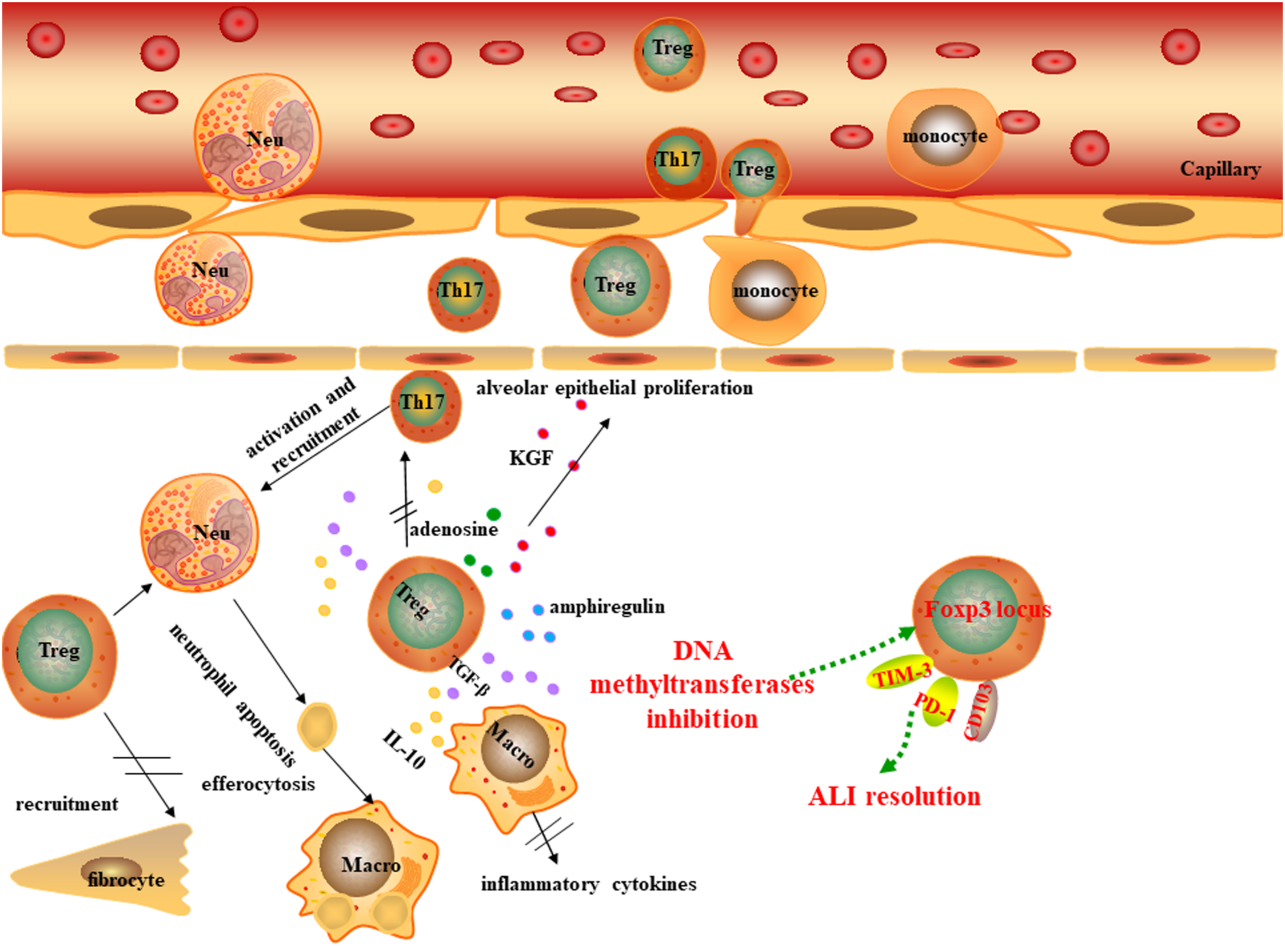
The potential mechanism of Treg suppress the inflammation and promote of resolution of ALI. Neutrophils, macrophages, and Th17 cells are recruited into alveola, and release inflammatory mediators lead to damage of endothelium, thus pulmonary edema during ALI. IL‐10 and TGF‐β secretion of Tregs can inhibit the proliferation and function of Th17 cells and macrophages. TGF‐β also can mediate apoptosis of neutrophil apoptosis and cytokines secretion of macrophage, promote the barrier repair. Tregs also can control of fibrocyte recruitment to the lung to inhibit the fibroproliferation. Tregs also can promote resolution of ALI through CD73‐dependent adenosine generation. Treg number and function can be partly enhanced by DNA methyltransferase inhibition to accelerate repair of lung injury. Moreover, Tregs exert tissue repair function by expression KGF and amphiregulin.

The main mechanisms of CD4^+^CD25^+^Foxp3^+^Tregs in alveolar to improve ALI resolution are mediated by inducing neutrophil apoptosis and suppression of macrophage anti‐inflammatory cytokine secretion^8^, ^72^; control fibrocyte recruitment to lung to inhibit the fibroproliferation though CXCL12‐CXCR4 axis^73^; CD73‐dependent adenosine generation.^74^ Singer group also found that the number and Foxp3 expression, activation state, suppressive phenotype, and proliferative capacity of CD4^+^CD25^+^Foxp3^+^Tregs in lung enhanced in mice treated by DNA methyltransferase inhibitor indicated that epigenetic pathways are very likely to be novel targets for the treatment of ARDS.^75^ Epithelial repair also plays important role in resolution of ALI. Data of Dial et al. indicated that Foxp3^+^Tregs can secret keratinocyte growth factor (KGF) to enhance alveolar epithelial proliferation.^76^ Moreover, Tregs also can directly exert tissue repair function, at least in part, through production of amphiregulin in influenza‐induced ALI model (Figure 3).^77^

## 5 FUTURE DIRECTIONS AND PERSPECTIVES

Clarifying the roles of Tregs in ALI resolution may lead to the design of new treatments for patients with ARDS. And therapies aimed at expansion or adaptive transfer of Tregs to ARDS patients could be an effective approach.

Studies have found leukotriene B4 Receptor (BLT1) and alanyl‐glutamine (Ala‐Gln) could recruit CD4^+^CD25^+^Foxp3^+^Tregs of alveoli. The blockade of LTB4‐BLT1 pathway significantly decreased Tregs numbers in BALF and impaired ALI resolution.^78^ And intragastric gavage Ala‐Gln could regulate the polarization of Tregs and Th17 cells to promote the resolution of lung inflammation.^79^ Moreover, our previous studies also indicated that lung‐resident mesenchymal stem cell can maintain balance of Tregs and Th17 cells and upregulate Treg/Th17 ratio.^80^ These data above tell us that finding ways to increase the number or function of Tregs or upregulate the Treg/Th17 cell ratio may be an effective way to alleviate lung injury and promote lung repair.

The adoptive transfer of regulatory lymphocytes to patients with ALI is a good idea. D'Alessio et al. found the transfer of Tregs significantly improved survival rate and resolution of ALI.^8^ But there are concerns that Tregs tend to exhibit remarkable plasticity. It remains unknown whether Tregs differentiate into effector T cells after adaptive transfer to ARDS patients. Moreover, therapeutic effect of Tregs depend on its relative contributions and the timing during the course of ALI initiation, pathogenesis and resolution. Up to now, there is only evidence of Tregs transfer to promote the resolution of ALI, but no studied pay attention to the timing of Tregs.

## 6 CONCLUSIONS

ARDS is a hard clinical problem with high mortality in critically ill patients. No specific therapies are available. Studies of ALI indicate that Tregs attempt to promote the resolution of ALI by regulating actively innate and adaptive immune responses. Identifying how to best isolate and augment Tregs in vivo or ex vivo and avoid Tregs depletion are critical aims that are the potential treatment of ALI/ARDS.

## CONFLICT OF INTEREST

The authors declare that they have no competing interests.

## ETHICS STATEMENT

The Ethics statement is not applicable in this review.

## AUTHOR CONTRIBUTIONS

Linlin Wang, Weipeng Jiang, and Xiaocen Wang involved in writing manuscript draft, searched for literatures, and contributed to final draft. Yuanlin Song and Lin Tong revised the manuscript. All authors have read and approved the final draft of the manuscript for publication.

## Data Availability

Research data are not shared.
